# Gold extraction at the molecular level using α- and β-cyclodextrins

**DOI:** 10.3762/bjoc.21.89

**Published:** 2025-06-06

**Authors:** Susana Santos Braga

**Affiliations:** 1 LAQV-REQUIMTE and Department of Chemistry, University of Aveiro, 3810-193 Aveiro, Portugal

**Keywords:** bromoaurate, cyanoaurate, cyclodextrin inclusion, self-assembly, supramolecular interactions

## Abstract

Cyclodextrins (CDs) are known for their ability to form supramolecular interactions with a wide range of guest molecules. In this review, focus is given on the inclusion of complex aurate ions such as tetrabromoaurate, dicyanoaurate and a few other tetrahaloaurates. The review describes the properties of self-assembly of cyclodextrins with these ions, with highlight to α-CD and, more recently, β-CD, requiring the use of a co-former/precipitating agent. Practical applications of this ability are directed to the selective isolation of gold from a variety of sources, spanning from gold-rich mining ores and tailings/mining wastes to gold-bearing metal scraps obtained from disposed electronic devices. Moreover, it describes the development of a method based on spontaneous complex formation between α-CD and tetrabromoaurate and its current status of use in a few mining sites in the United States.

## Introduction

Gold has allured and captivated humankind ever since its discovery, mostly because of its glittery metallic yellow color and its ability to remain untarnished even after exposure to extremely aggressive conditions. Gold is able to keep its shine for centuries, having been deemed as the most noble of metals [1]. Present in human life since early civilizations, its use across various ancient societies can be traced back to more than 7000 years ago, as evidenced by the artefacts of goldsmiths crafts found in Minoic, Egyptian, and South American tombs [2].

In the present day, the uses of gold have grown beyond minting, jewellery, and ornaments, much because of its unique properties. Gold is soft, ductile, malleable, and a good conductor of heat and electricity, having the lowest electrochemical potential amidst metals. This means that cationic gold (in any form) can accept electrons from any reducing agent to form metallic gold while, in turn, metallic gold is easily reduced to Au^−^ [2]. Adding to the aforementioned properties, the excellent resistance against corrosion makes gold a vital element for use in a wide range of electronic applications. Some common devices that contain gold include smartphones, in which it is used in the circuit boards and connectors to ensure good conductivity and durability [3–5], computers and laptops, which incorporate it in components such as RAM (random access memory) modules [5–6], processing units, and printed circuit boards [5]. Small amounts of gold are also used in some connectors/cables for video and/or audio, bringing the benefit of enhanced signal transmission (because of its high conductivity), reliability and durability (a direct result of its high resistance to oxidation) [7–8]. In medicine, gold is used as an inert and non-allergenic alternative metal in devices such as pacemakers [9–10] and a variety of implants, from hearing aids to prosthetic coatings and endovascular stents [11]. Traces of gold are found in a few electronic components of the automotive industry, such as airbag sensors, circuit boards, and audio systems [12]. Last but not least, gold coatings have gained a relevant role in aerospace technology, being used in satellites [13] and telescopes [14] to protect sensitive components against the damaging effects of solar radiation.

Production of gold encompasses the mining stage, followed by one more processing steps. Post-extraction processing can be carried out by several methods, with highlight to gravity concentration (based on the higher density of gold), cyanidation, and mercury amalgam [15]. The gravity method is a simple and, from the viewpoint of the environment, relatively harmless process that works well as a stand-alone for high-density gold particles and alluvial deposits. In 2004, it accounted for 20 to 25% of the global gold production [15]. The gold–mercury amalgam method is employed by small-scale and artisanal gold producers, accounting, in 2011, for 17–20% of the global gold production [16]. As denoted by its name, the process uses metallic mercury to extract gold from ore as a stable amalgam. Following, the amalgam has to be oven-heated or torch-burned to allow mercury to sublimate away from the gold. This causes significant health impacts on human and wildlife due to mercury gas inhalation, and on the surrounding ecosystems due to mercury accumulation in soil and water [17].

The shortage of natural resources in tandem with the increasing market demand for gold are driving the need to obtain this commodity from low grade ores, typically containing less than 1 wt % in gold, materials of difficult extraction, such as the gold-bearing silicates and even non-ore sources (e.g., mining wastes) [18] and abandoned electrical and electronic equipment, called e-waste, which can contain up to ten times more gold than waste ores [19]. To retrieve gold from these materials, the most widespread process is to use potassium or sodium cyanides, which are the most effective solvents for gold [20]. Because of the high toxicity and strong environmental impact of cyanides, eco-friendlier alternatives are currently under research. Some are based on replacing cyanide with less harmful chemicals, such as thiocyanate, thiourea, or thiosulfate. Another alternative is the development of microbial leaching methods [18]. These resort to either cyanogenic microorganisms, such as *Chromobacterium violaceum*, *Pseudomonas fluorescens,* and *Pseudomonas plecoglossicida*, which incorporate gold into their unicellular bodies when grown in the presence of gold-containing e-waste [21], or to acidophilic microbes such as chemolithotrophic bacteria and archaea, which can accelerate the oxidative dissolution of gold sulfides in a biotechnological extraction process suited for sulfidic ores [22]. Microbial leaching methods are not, however, implemented at the industrial scale, which still depends solely on the highly pollutant cyanide leaching process.

An emerging strategy for gold recovery is through supramolecular chemistry, with studies indicating that macrocycles such as 18-crown-6 [23], cucurbit[6]uril [24], cucurbit[8]uril [25], and cyclodextrins can isolate gold with high selectivity. The present review is centered on the use of cyclodextrins, natural and biocompatible oligosaccharides that form upon microbial fermentation of starch. The method is environmentally safe because it resorts to natural compounds, posing as a promising alternative to the use of cyanides and mercury. For now, let us look at what cyclodextrins are and which properties make them so special and versatile for use in a variety of industrial processes. Originating from starch – a helicoidally shaped glucose chain – cyclodextrins are small fragments that are sectioned off and linked back together to form cyclic oligosaccharides. The naturally occurring cyclodextrins, also named native cyclodextrins, have typically six (α-CD), seven (β-CD) or eight (γ-CD) ᴅ-glucose units [26–27]. Their monomers are all linked by α-1,4 glycosidic bonds, which results in a molecular geometry with a shape that resembles a truncated cone. Secondary hydroxy groups of the glucose residues are directed towards the wider rim of the cone, and primary hydroxy groups, bound to C6, face the narrower rim, which overall conveys them with good aqueous solubility. The inner cavity of the cone is, in turn, lined with protons, which makes it sufficiently hydrophobic to host apolar guest molecules, forming particular adducts called inclusion compounds. One obvious consequence of inclusion is increased solubility, with another important action being the protection of the included guests against degradation from light, heat, oxidation, and hydrolysis. Owing to their properties, cyclodextrins are frequently applied in pharmaceutical formulations with low-soluble or unstable drugs [28–30], in cosmetic formulations [31–32], and in the food industry [33–34]. The vast majority of guests in these applications are organic compounds, with the driving forces leading to inclusion being van der Waals and hydrophobic interactions for apolar guests, and/or hydrogen bonding when the guest features H-bond accepting groups that interact with the hydroxy groups of the cyclodextrins. Interactions with metal ions/complexes and organometallics guests, albeit less common, are also observed. These form a variety of structures from supramolecular second-sphere coordination adducts to pseudorotaxanes [35–36]. With highly charged metal ions, electrostatic interactions and first-sphere coordination bonds are dominant, while second-sphere coordination with inorganic and organometallic complexes involves hydrophobic interactions and van der Waals forces (mainly with the organic parts of these guests).

Several forces are at play in the formation of supramolecular assemblies with gold in the form of haloaurate ions, as detailed in the forthcoming sections of this review. The combination of interactions linking cyclodextrin channels with infinite polymer chains of tetrahaloaurate ions and their counterions affords a highly specific and stable superstructure that is the working pillar for the efficient and selective process of gold recovery from complex matrices such as ores and industrial waste.

## Review

### Isolating gold with α-cyclodextrin

#### Process discovery and the role of self-assembly

**Figure 1:**
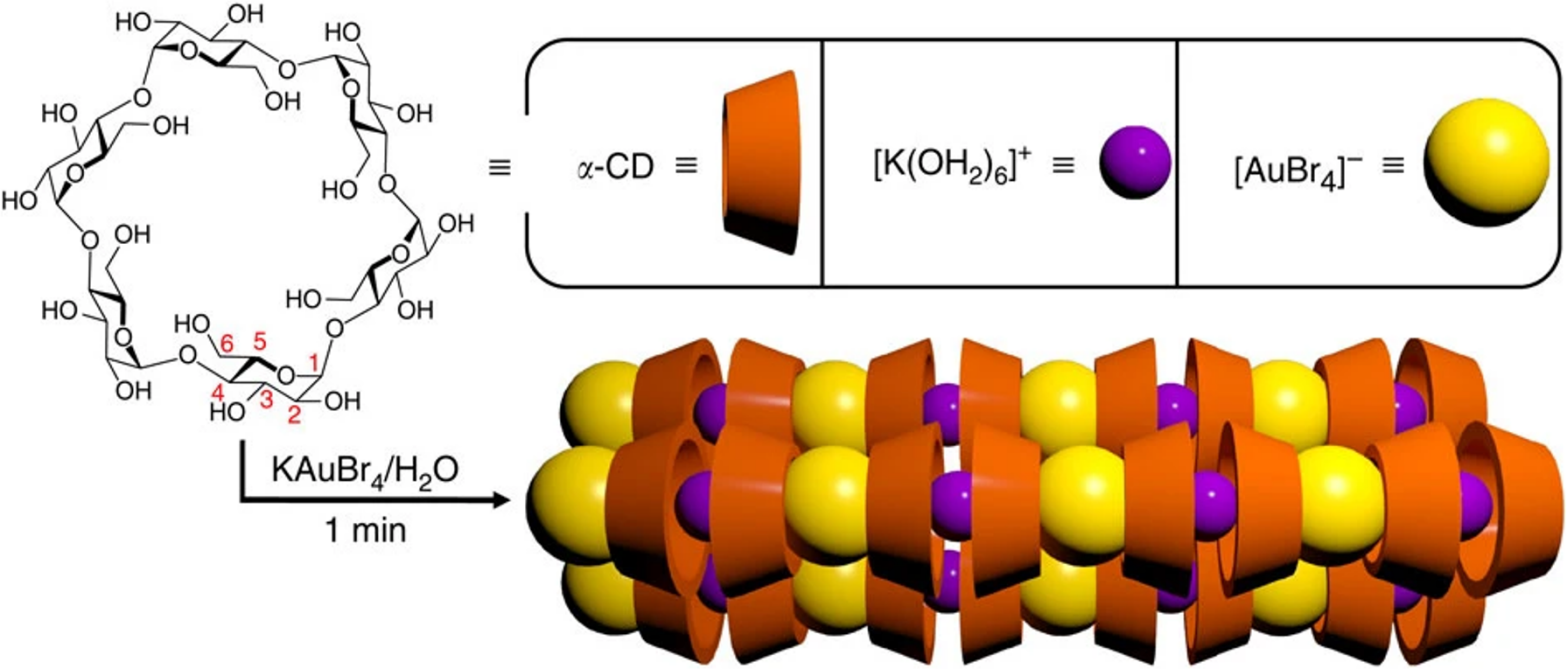
Schematic depiction of the α-CD channels containing the polyionic {[K(OH_2_)_6_]^+^[AuBr4]^−^}*_n_* chain inside. Reproduced from [37] (“Selective isolation of gold facilitated by second-sphere coordination with α-cyclodextrin“, © 2013 Z. Liu et al., published by Springer Nature, distributed under the terms of the Creative Commons Attribution-NonCommercial-NoDerivs 3.0 Unported License, http://creativecommons.org/licenses/by-nc-nd/3.0). This content is not subject to CC BY 4.0.

The discovery that α-CD could be used to isolate gold was made serendipitously [37]. A young researcher of the Stoddart group, Zhichang Liu, was trying to prepare tridimensional γ-CD metal-organic frameworks (CD-MOFs) [38–39] using a variety of salts instead of the traditional KOH employed to link them together. In his experiments, he used a different cyclodextrin, α-CD, in the place of the traditionally employed one, γ-CD. α-CD molecules did not, however, form the expected tridimensional CD-MOF in the presence of KAuBr_4_ in aqueous solution. Instead, they self-assembled into unidimensional threads that aggregated into superstructures and formed, in less than one minute, needle-shaped crystals visible to the naked eye. In these crystals, the molecules of α-CD stacked by hydrogen-bond interactions and oriented in alternating head-to-head and tail-to-tail modes comprising a continuous channel filled by an alternating [K(OH_2_)_6_]^+^ and [AuBr_4_]^−^ polyionic chain (Figure 1).

The authors further demonstrated that the process is selective for gold by determining, using ICP-OES, the precipitation ability of α-CD (0.2 mmol × 2) of a solution containing gold in the presence of palladium and platinum (33 mM KAuBr_4_, 33 mM K_2_PdBr_4_, and saturated K_2_PtBr_4_, at ≈26 mM), with 78.3% of the gold precipitating out of solution while for the other metals there was only precipitation of trace amounts (<3%).

**Figure 2:**
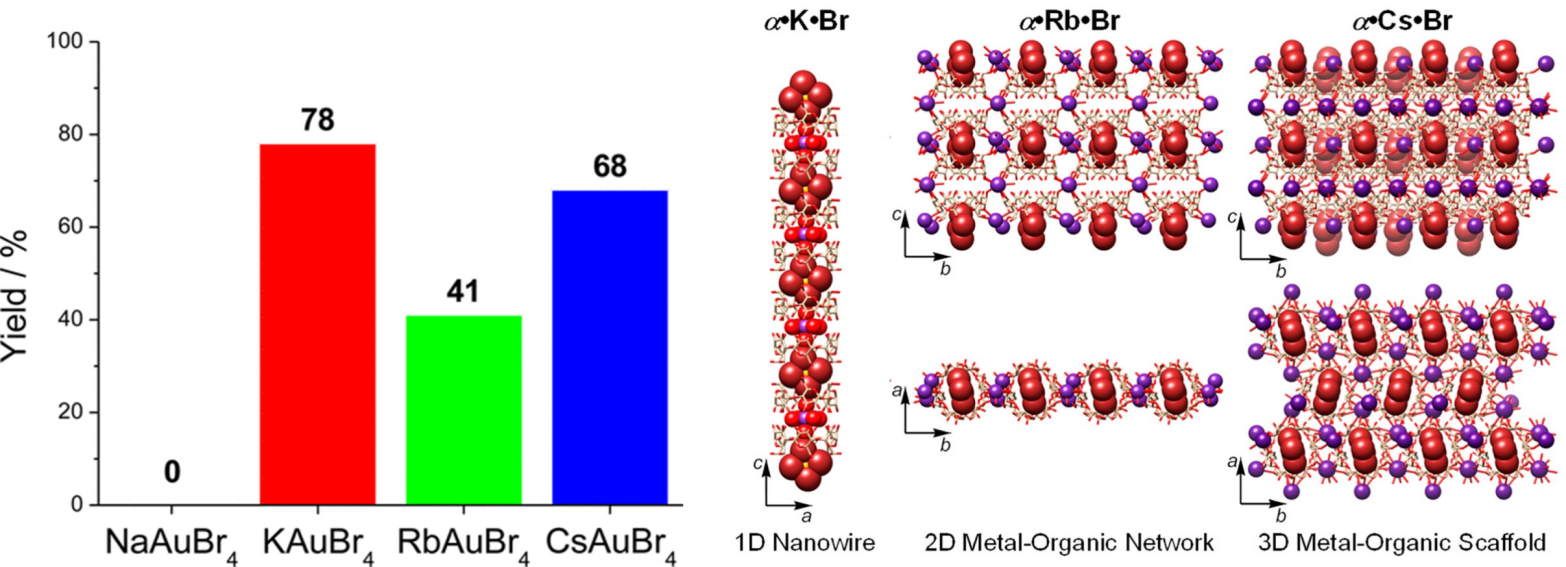
Complexes of α-CD with MAuBr_4_ salts. Left) Co-precipitation yields from aqueous solutions of α-CD (20 mM) and MAuBr4 (10 mM) (M = Na/K/Rb/Cs) measured at 20 °C by UV–vis spectrophotometry. Right) Structures of the adducts present in co-precipitates with different alkali metals. Reproduced from Z. Liu et al. [40], “Cation-Dependent Gold Recovery with α-Cyclodextrin Facilitated by Second-Sphere Coordination”, *J. Am. Chem. Soc*., © 2016 American Chemical Society, distributed under the Standard ACS AuthorChoice/Editors’ Choice Usage Agreement, https://pubs.acs.org/page/policy/authorchoice_termsofuse.html. This content is not subject to CC BY 4.0.

Investigations on the role of both the halogen in KAuX_4_ (X = Br, Cl) [37], as well as of different alkali cations (Na, K, Rb, Cs) [40] upon the ability to form a co-precipitate in the presence of α-CD were undertaken. The possibility of using different cyclodextrins (α-CD, β-CD or γ-CD) for precipitation was also evaluated [40]. When another halogen was used, no precipitation was observed between α-CD and the KAuCl_4_ salt [37]. When CDs with a larger cavity were tested (β-CD and γ-CD), gold particles were dissolved but there was no immediate precipitation [40], thus highlighting the importance of a host–guest tight fit to obtain a precipitate with KAuBr_4_, which occurs only with α-CD. Lastly, tests with alternative alkali metals showed that two of the salts were able to afford co-precipitates with α-CD, as shown in Figure 2.

In the adducts with the large-diameter alkali metal cations, the geometry is different from that observed in {α-CD·[K(OH_2_)_6_]^+^[AuBr_4_]^−^}*_n_*, because coordination of Rb^+^ and Cs^+^ takes place both at OH groups and at the O atoms of the glucopyranosyl ring in the CD tori (Figure 2, right). This strong coordination, in cooperation with the geometrically favourable second-sphere coordination of [AuBr_4_]^−^ and two α-CDs, accounts for the co-precipitation of α-CD·[Rb(OH_2_)_6_]^+^[AuBr_4_]^−^ and α-CD·[Cs(OH_2_)_6_]^+^[AuBr_4_]^−^.

Improvements of the method for the precipitation of gold in the form of {α-CD·[K(OH_2_)_6_]^+^[AuBr_4_]^−^}*_n_* were further investigated, namely the effect of temperature and ratio of α-CD that was added to the salt solution [40]. Studies at different temperature values demonstrated that the precipitate is less soluble as the temperature decreased and thus the gold extraction was optimal at 0 °C. Tests with excess stoichiometries of α-CD (2:1, 3:1, 4:1, 5:1) showed that a 2:1 ratio (α-CD to KAuBr_4_) was sufficient to ensure a co-precipitation yield of 78% and that this value remains relatively constant with increasing amounts of α-CD. It was, thus, concluded that adding extra amounts of α-CD do not help increasing the yield and that the co-precipitated adduct has the constant composition of 2:1 for α-CD to KAuBr_4_, in consistency with the single-crystal X-ray data.

#### Application studies in gold mining facilities

**Process patenting and scale-up pilot trials in the United States:** The discovery that α-CD can quickly precipitate gold, as detailed in the previous subsection, has sparkled the interest for industrial applications ranging from the recycling of gold-bearing e-waste [41] to the isolation of gold from mining sites.

In 2014, a new company, Cycladex, was funded by Sir Fraser Stoddart and Dr. Roger Pettman with the mission of conducting eco-friendly mining of precious metals [42]. The process of gold precipitation with cyclodextrins, bromic acid and potassium hydroxide to form the potassium bromoaurate salt was patented, with patent coverage expanding to metals like silver, platinum, palladium, and rare earths, as well as the use of other cyclodextrins, β- and γ-CDs, other acids (including hydrogen halides, nitric acid, sulfuric acid, and mixtures thereof) and sodium hydroxide (instead of potassium hydroxide) [43].

Scale-up pilot trials on 20 batch samples were conducted in Arizona with partnership from companies already active in the market. Results revealed excellent recoveries and showed that the cyclodextrin-based leaching agent can be continuously recycled. Moreover, the time needed for leaching was shorter: extraction with the conventional cyanide process took 30 hours, while the cyclodextrin-based process was done in 4–5 hours [44]. Currently, the process is being applied in Cycladex facilities in Nevada and licensed for use by other companies around the globe [42].

**Tests on ores from African mines:** In 2020, a variant of the α-CD precipitation method was developed with the aim of acting as a direct purification step for batches of gold ore [45]. The ores were obtained in an artisanal way from the Shanono gold deposit in Kano State, Nigeria. The researchers involved in this study used a modified aqua regia solution to digest the ore concentrate, in which hydrochloric acid was replaced by hydrobromic acid. Upon addition of α-CD and KOH, there was the formation of the {α-CD·[K(OH_2_)_6_]^+^[AuBr_4_]^−^}*_n_* complex, leading to immediate precipitation. Isolation of metallic gold from the precipitate was achieved by addition of an organic reducing agent. Moreover, the study optimized the method to yield as much as 69% of gold from the original ore. This was almost twice the 35.5% yield obtained with the mercury–amalgam method, which is currently in use by Artisan Small Scale Miners. Artisan Small Scale Miners is an association of familial and small-scaled entities across a range of countries located mainly in Africa, Asia, and South America [46].

A few years later, the process of gold ore purification was further optimized to achieve yields as high as 98.9%. Using ores from Democratic Republic of Congo (initial sample mass of 400 g, acqua regia dissolution) researchers used a combination of experimental measurements (of yield) and neural networks to optimize the chemical parameters of the extraction. An optimal value of gold removal, lying between 98 and 98.9%, was found to occur with a contact time of 40 minutes, a concentration of α-CD of 11.61 g/L and KOH was added in enough quantity to raise the solution pH to 5 [47–48].

#### Complexes of α-CD and cyanoaurate

A commonly used step in cyanide leaching is to pass gold-bearing cyanide solutions through activated carbon beds to help concentrate the gold. Following this step, the gold that adsorbed onto carbon is removed either by aggressive solutions (cyanide or hydroxide leaching), or by processes with high energetic costs such as heating or applying high pressure. Seeking to find an eco-friendlier method to strip the gold from carbon, the Stoddart group investigated the use of α-CD in a series of tests at the laboratory scale in which suspensions of KAu(CN)_2_-loaded carbon (50 mg, containing 0.6 mg of gold) were mixed for 30 minutes with increasing amounts of α-CD (concentrations ranging from 1–10% w/v) [49]. Extraction yields were shown to increase with concentration of α-CD. The highest concentration tested, 10%, afforded 19% yield in gold. Competitive extraction tests with gold and silver in solution showed that not only the striping process was quite selective for gold, but also that the presence of silver helps to increase the yield of gold extraction to 31% (with 10% α-CD). This result was attributed to a competing effect of KAg(CN)_2_ on the carbon surface that promoted the desorption of KAu(CN)_2_.

**Figure 3:**
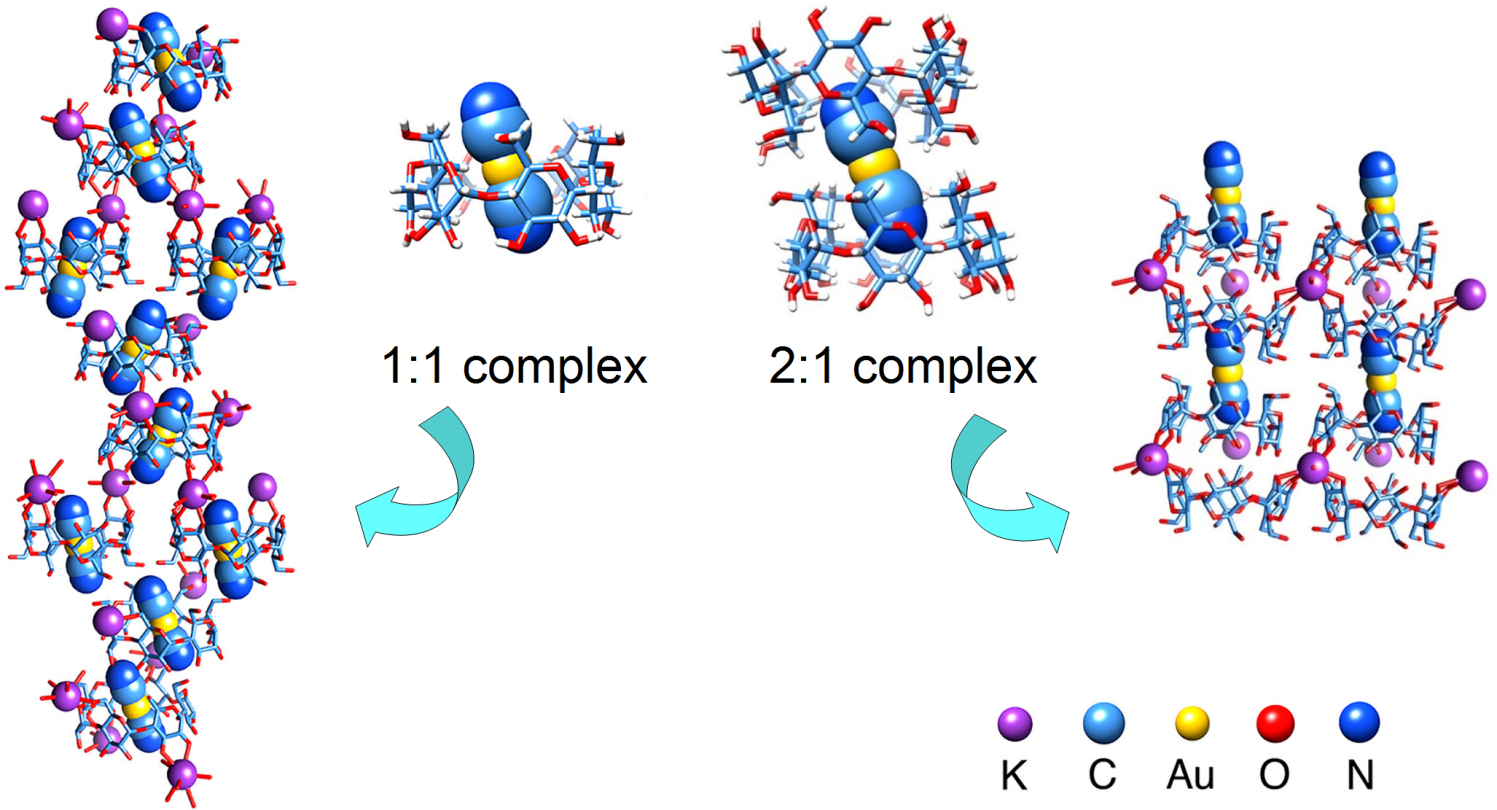
Crystal structures of the complexes of α-CD with KAuCN_2_ salts, with tubular representation for α-CD and space-filling for KAu(CN)_2_. The images at the center depict the interaction between one Au(CN)_2_^−^ anion and one or two α-CD(s), according to the stoichiometry. The images at the sides depict the packing for the 1:1 (left) and the 2:1 (right) complexes, showing the positions of K^+^ cations and Au(CN)_2_^−^ anions as well as the relative dispositions of the α-CD tori. Adapted with permission from [49], Liu, W.; Jones, L. O.; Wu, H.; Stern, C. L.; Sponenburg, R. A.; Schatz, G. C.; Stoddart, J. F. Supramolecular gold stripping from activated carbon using α-cyclodextrin. *J. Am. Chem. Soc*. **2021**, *143*, 1984–1992. Copyright 2020 American Chemical Society. This content is not subject to CC BY 4.0.

The ability of α-CD to strip gold from carbon was shown to result from the formation of inclusion complexes directly with the cyanoaurate ions, with (at least) two different stoichiometries of α-CD:KAu(CN)_2_, 1:1 and 2:2. Structural elucidation was achieved by isolation and X-ray diffraction of single crystals (Figure 3), showing, for both 1:1 and 2:1 stoichiometries, cyanoaurate anions inside the host cavity and potassium cations interacting with hydroxy groups of adjacent cyclodextrins.

The strong host–guest affinity and complex stability in the solution phase has been studied by several methods, with ^1^H NMR titrations revealing a 1:1 stoichiometry in D_2_O (thus, 1:1 being preferential in solution). The binding constant (*K*_a_) between Au(CN)_2_^−^ and α-CD in D_2_O was determined to be 8.1 × 10^4^ M. Titration calorimetry produced a binding constant value of 1.5 × 10^4^ M for the 1:1 complex, thus confirming a strong interaction affinity lying within the 10^4^ molar range.

### Gold extraction with β-CD and derivatives

The possibility of using β-CD instead of α-CD in gold recovery and extraction is highly sought after by both researchers and industry because of the readiness of availability of β-CD, its low price, and its versatile chemistry. The studies described in this section have focused their application on the extraction of gold from electronic waste, or e-waste, which can be an important source of gold as mentioned in the introduction. For example, a ton of discarded mobile phones can yield 150 grams or more of gold, according to a study conducted in Japan in 2008 by recycling companies [50]. It is important, however, that the gold extraction method for e-waste sources is affordable in order to hold up the interest in the recovery, separation, and recycling of these materials. A common process of extraction of gold from e-waste is through acid leaching (typically with acqua regia), in which gold is dissolved in the form of tetrachloroaurate anions (AuCl_4_^−^). Subsequently, gold is precipitated upon conversion to its metallic form (Au^0^) using an affordable reducing agent such as sodium sulfite. As a disadvantage, the step of gold reduction causes co-precipitation of all other metals that are present in solution, which has raised the need for the use of selective precipitation methods. One of the strategies to address this selectivity precipitation issue is the use of β-CD.

#### Precipitating β-CD:KAuBr_4_ with the help of a co-former

The studies conducted within the Stoddart group in 2016 had shown that, albeit β-CD was able to interact with KAuBr_4_ in aqueous solution and form inclusion complexes [40], the complex did not fall out of solution in the form of a precipitate. Instead, slow evaporation of the aqueous solvent was required in order to obtain the complex as a crystalline solid. This lengthy isolation made the use of pure β-CD unsuited for industrial application in gold recovery, driving research on co-precipitation methods – these resort to the addition of a co-former, that is, a substance that causes the precipitation of the product as a solid.

**Figure 4:**
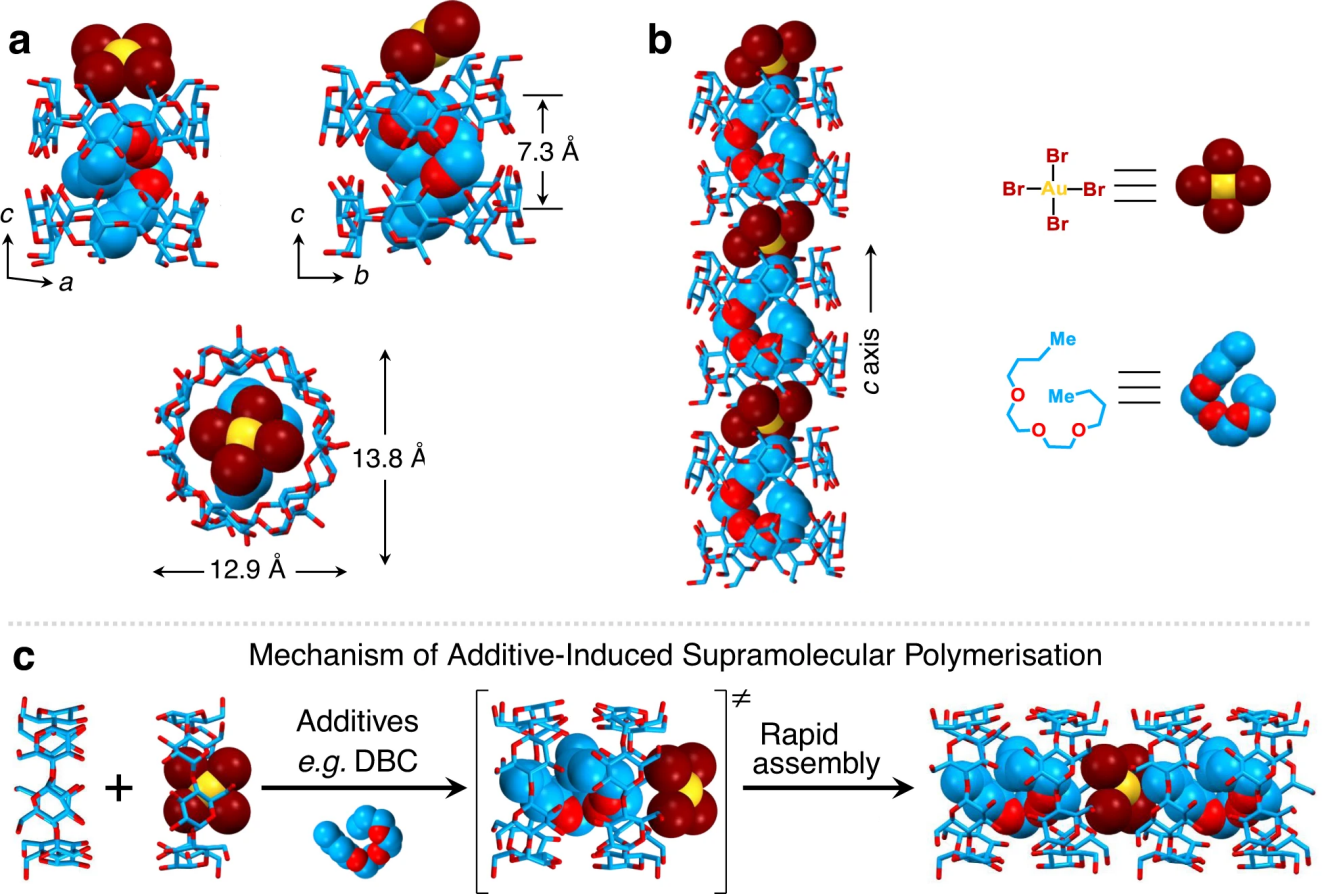
Solid-state structure of the complex 2β-CD·HAuBr_4_·DBC. (a) Capped-stick and space-filling representation of different views of 2β-CD·HAuBr_4_·DBC, showing the packing mode and dimensions of the complex. (b) Capped-stick and space-filling representation of the β-CD channels extending along the *c*-axis, which are occupied by alternating DBC and [AuBr_4_]^−^ anions. Solvent molecules and hydrogen atoms have been omitted for the sake of clarity. C skyblue, O red, Br brown, Au yellow. (c) Schematic illustration of the mechanism leading to precipitation (named “supramolecular polymerisation” by the authors) after adding various additives to the solution of β-CD and [AuBr_4_]^−^ anions. Reproduced from [51] (© 2023 H. Wu et al., published by Springer Nature, distributed under the terms of the Creative Commons Attribution 4.0 International License, https://creativecommons.org/licenses/by/4.0).

In this context, the addition of anti-solvents to aqueous solutions is a common strategy in precipitate formation. In 2023, the same research group showed that various solvents, some miscible with water and others not, led to the quick formation of co-precipitates when added to aqueous solutions of β-CD and KAuBr_4_ (both at 5 mM): dibutyl carbitol, isopropyl ether, chloroform, dichloromethane, hexane, benzene, and toluene [51]. The most effective one was dibutyl carbitol (DBC), which caused 99.7% precipitation when added to reach a final concentration of 0.25% (v/v). To better understand the role of DBC, crystals of the ternary complex β-CD·KAuBr_4_·DBC were obtained by slow vapor diffusion of DBC into an aqueous solution of KAuBr_4_ and β-CD over three days. Single-crystal diffraction showed the [AuBr_4_]^−^ anion centered between the primary faces of two adjacent β-CD molecules, while DBC was nested inside the barrel-shaped cavity of a β-CD head-to-head dimer (Figure 4). Noteworthy, K^+^ ions were absent in the crystal superstructures, which was explained by the authors as the possible result of protons replacing K^+^ ions during crystallization.

Aiming at developing a protocol for gold recovery from e-waste and gold-bearing scrap based on the use of β-CD, extraction parameters such as the ratio of β-CD to KAuBr_4_, and the countercation of the gold salts were explored [51]. When the range of the β-CD-to-KAuBr_4_ ratio increased from 0.5 to 2.5, the percentage of gold also increased, showing a quasi linear dose-dependent relation and gold precipitation yield reaching 99.0% for the 2.5 ratio. Regarding the countercation, no effect on gold precipitation was shown, gold-recovery efficiencies being 97.5% with NaAuBr_4_ and 98.5% with HAuBr_4_ (i.e., practically identical to that of KAuBr_4_).

The process of recovery was tested at the laboratorial scale on a spent gold-bearing alloy cable obtained from a local electronic junk shop. The cable was leached with HBr/H_2_O_2_ to obtain a solution of HAuBr_4_, which was treated with a saturated solution of β-CD and DBC at 0.1% (v/v) and stirred for five minutes to ensure maximal gold precipitation. Conversion of the [AuBr4]^−^ anions trapped in the co-precipitate to gold metal was done by adding a reducing solution of N_2_H_4_·H_2_O. The process further allowed recycling β-CD from the solution used to wash the gold precipitate by using the anti-solvent acetone.

#### Thiolated β-CD derivative for selective gold precipitation

**Figure 5:**
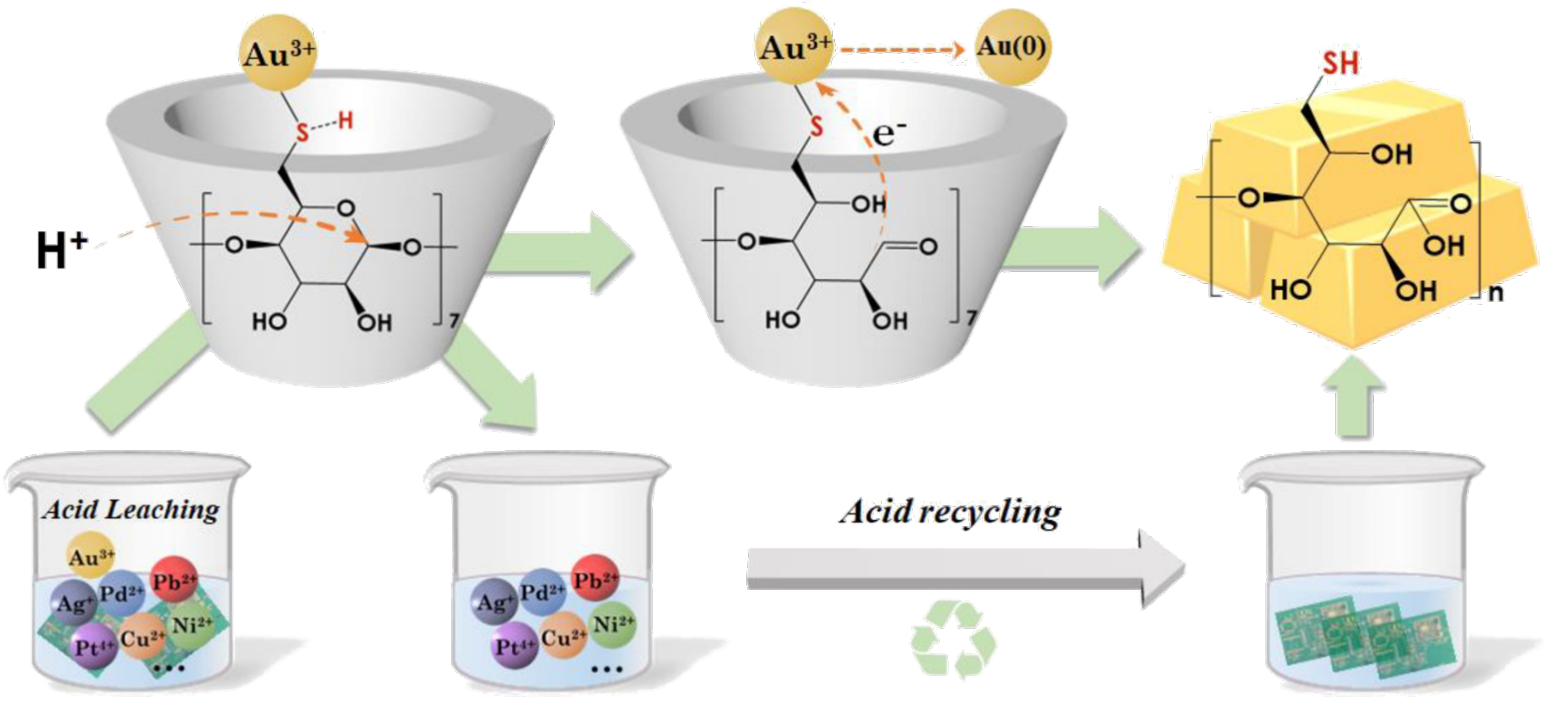
Schematic depiction of the selective removal of AuCl_4_^−^ and its precipitation as solid gold from e-waste acid leachates using a thiolated β-CD derivative. Reproduced from [52], *Resources, Conservation and Recycling*, vol. 193, by D. Fang; F. Bi; L. Yang; W. Hu; Z. Hua; H. Wei; C. Chen; Y. Tu; P. Shao; M. Li; X. Luo; G. Yang, “Selective extraction of gold from strong acid e-waste leachate through H^+^-triggered cascade reaction of thiolated β-cyclodextrin”, article no. 106958, Copyright (2023), with permission from Elsevier. This content is not subject to CC BY 4.0.

Fang et al. reported the synthesis and application of a 6-thiolated β-CD derivative (with unspecified degree of substitution) to precipitate metallic gold from strongly acidic leachates (pH < 2) [52]. To better understand the chemistry behind this precipitation method, it is worth mentioning that the AuCl_4_^−^ anion is a complex ion, formed by gold(III) and chloride ions, and that the presence of the extra choride ion to render it an anionic complex instead of a salt is a factor that contributes to the overall aqueous solubility and stability of AuCl_4_^−^. The precipitation method, according to the authors, is postulated to involve acid hydrolysis of the acetal group of thiolated β-CD to form a reactive aldehyde that takes part in the reduction of the Au^3+^ cations that get bound to the thiol groups in the cyclodextrin. The authors further reported the process to be selective and to have good efficiency, with a gold removal yield as high as 96.1% in the presence of the thiolated β-CD derivative (Figure 5). Cyclodextrin recyclability was, however, not demonstrated and it is expectedly unfeasible because it suffered acid hydrolysis during gold precipitation reaction.

## Conclusion

The present review describes the available literature reports on the use of α- and β-cyclodextrins for sustainable isolation of gold. The majority of reports, as well as the developed processes of recovery, revolve around the ability of α-CD·bromoaurate complexes to form infinite channels that self-aggregate and spontaneously precipitate as needle-shaped crystals. The discovery, serendipitous in its essence, has opened a new area of research and ended up bringing significant impact on the gold mining industries. A gold recovery method based on the use of α-CD is patented and in use in the present day. It has as advantages reduced extraction time when compared to the traditional method of cyanide leaching, low environmental impact, and enhanced recovery rates. Stemming from this research, niche applications of α-CD to specific processes of the gold extraction process, such as its removal from carbon adsorbents that help concentrate it when starting from low yield raw materials, have emerged. In gold stripping from carbon beds, the use of α-CD results in the formation of a new supramolecular complex, α-CD·cyanoauride. Moreover, the use of α-CD offers the benefit of a selective isolation of Au(CN)_2_. The path is open for the integration of the α-CD stripping method into commercial gold mining protocols, with expected reductions in costs, energy consumption, and environmental impact.

Research on the gold-precipitating properties of a more affordable cyclodextrin, β-CD, is, on the other hand, still taking its first steps. In the case of the bromoaurate ion, the method implies the use of a precipitate co-former. There are, nonetheless, advantages to the use of additives, namely the possibility of precipitating gold from a solution even when it is found in low concentrations and the possibility of operating at ambient temperature. Lab-scale tests with e-waste recovery show promising extraction yields. Future developments will further optimize parameters and scale up the procedure envisioning its expansion to industrial application in the recycling of gold-bearing e-waste.

## Data Availability

Data sharing is not applicable as no new data was generated or analyzed in this study.
